# Recognition of Emotion Intensities Using Machine Learning Algorithms: A Comparative Study

**DOI:** 10.3390/s19081897

**Published:** 2019-04-21

**Authors:** Dhwani Mehta, Mohammad Faridul Haque Siddiqui, Ahmad Y. Javaid

**Affiliations:** Electrical Engineering and Computer Science Department, The University of Toledo, 2801 W Bancroft St, MS 308, Toledo, OH 43606, USA; dhwani.mehta@utoledo.edu (D.M.); mohammadfaridulhaque.siddiqui@utoledo.edu (M.F.H.S.)

**Keywords:** automatic facial emotion recognition, intensity of emotion recognition, behavioral biometrical systems, machine learning

## Abstract

Over the past two decades, automatic facial emotion recognition has received enormous attention. This is due to the increase in the need for behavioral biometric systems and human–machine interaction where the facial emotion recognition and the intensity of emotion play vital roles. The existing works usually do not encode the intensity of the observed facial emotion and even less involve modeling the multi-class facial behavior data jointly. Our work involves recognizing the emotion along with the respective intensities of those emotions. The algorithms used in this comparative study are Gabor filters, a Histogram of Oriented Gradients (HOG), and Local Binary Pattern (LBP) for feature extraction. For classification, we have used Support Vector Machine (SVM), Random Forest (RF), and Nearest Neighbor Algorithm (kNN). This attains emotion recognition and intensity estimation of each recognized emotion. This is a comparative study of classifiers used for facial emotion recognition along with the intensity estimation of those emotions for databases. The results verified that the comparative study could be further used in real-time behavioral facial emotion and intensity of emotion recognition.

## 1. Introduction

The dual fears of identity theft and password hacking are now becoming a reality, where the only hope of a secure method for preserving data are behavioral systems. Systems which are based on user behavior are usually understood as behavioral systems. Behavioral traits are almost impossible to steal. Multiple commercial, civilian, and government entities have already started using behavioral biometrics to secure sensitive data. One of the major components of behavioral biometrics is the recognition of facial emotion and its intensity [1,2,3]. In the industry and academic research, physiological traits have been used for identification through biometrics. Any level of biometrics could not be performed without good sensors, and when it comes to facial emotion intensity recognition, apart from high-quality sensors (cameras), there is a need for efficient algorithms to recognize emotional intensity in real time. With the increased use of images over the past decade, the automated facial analytics such as facial detection, recognition, and expression recognition along with its intensity has gained importance and are useful in security and forensics. Components such as behavior, voice, posture, vocal intensity, and emotion intensity of the person depicting the emotion, when combined, help in measuring and recognizing various emotions.

**Figure 1:**
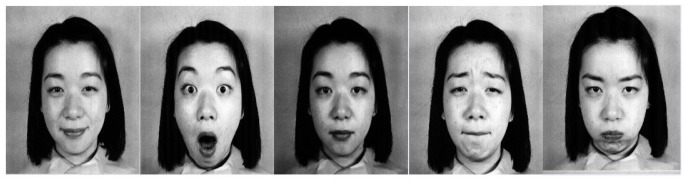
Illustration of 5 Basic Emotions: Happy, Surprise, Neutral, Sad and Angry.

Considering human facial images, as seen in Figure 1, recognizing the emotions and finding their intensity are vital. Primarily, 3D facial images are the most thoroughly researched [4,5,6,7] and predictions are made by the available systems based on the features that were extracted from the images for emotion and intensity recognition. The intensity of emotion plays a significant role in behavioral biometrics for future crime prediction systems. The intensity may be referred to as “*the degree of manifestation along the dimension of behavior*” [3]. The intensity of emotion is often directly associated with the intensity of facial muscle movements. This, in turn, indicates that the intensity of muscle movement represents the index of the intensity of emotional state, implying the intensity of the emotion that is being experienced. Such intensities can be measured in both spontaneous and posed expressions. These intensities are affected by the behavior of the person, whether the emotion depicted is voluntary or involuntary. Spontaneous facial expressions of an emotion indicate the behavior of the face that occurs when a person displays involuntary emotion, with no prior planning or intention. Posed facial expressions of emotions, on the other hand, are used on a large-scale for studies involving the intensity of facial emotions. The criterion for the accuracy of intensity detection of the five observed basic emotions (and a neutral expression) is based on the analysis of the facial behavior components that are relevant to emotional intensity communication [8]. This involves detecting the face and recognizing the intensity of emotion depicted, both of which could be achieved using classifiers assisted by a training set. Existing work gives a detailed survey of the algorithms used in this area [9,10,11]. Also, multiple intensities are measured with a value of rank one recognition and rank five recognition, where rank one is that the intensity is measured at the highest accuracy level and rank five at the lowest. Algorithms that have been used for feature extraction span from classical techniques such as principal component analysis (PCA) and linear discriminant analysis (LDA) to modern techniques such as machine learning (ML) and artificial neural networks (ANN) [10,11,12,13,14,15,16].

In recent years, major studies were carried out in the field of emotion intensity detection relating to three major areas. The first area relates to the cross-cultural character, where studies concluded that cultures highly agree with each other in facial emotion identification [8,17,18]. These studies also revealed a cross-cultural agreement in the prediction of the intensity of two different expressions for the same emotion [19]. The second area brings forward the research which shows that there exist major differences in gender skills to decode/predict nonverbal signs. In these studies, women have been superior predictors of emotions than men [20,21,22,23]. In the third area, several studies have been conducted, based on the five basic emotions, to find major error patterns and effects of the emotional intensity in diseases such as schizophrenia, autism, and borderline personality disorder [24,25,26].

Emotion recognition is being put to service in diverse real-life applications where a person’s emotional state serves as a cue to the successful operation of these systems. Physically and mentally challenged people are impuissant to show their emotions and require an alternate criterion to perceive their emotional state of mind. The Autism Spectrum disorders in an individual have been a core area of research in affective computing, and several research works have laid a tangible emphasis of emotion recognition for such cases [27,28,29]. Patients seeking therapies [30,31] and counselling for depressions [32], disorder of consciousness [33], and schizophrenia [34] have been a subject of interest in many explorations to unearth their curbed and concealed emotions. The information filtering systems also referred to as recommender systems, find an interest in detecting a person’s emotional state. These systems predict the preference of the user for a particular good or service and recommend the best possible option according to its ability. There are health-related recommender systems such as emHealth [35], hospital recommender systems [36], multimedia recommender systems [37,38] and for movies [39,40], web search [41] and e-commerce [42,43]. Prominent use of emotion recognition is in marketing and for getting automatic feedback. The concept, referred to as emotional marketing [44] milk affective computing for decision support [45,46] and for product feedback assessment [47,48]. E-Learning is also setting its feet wet by exploiting emotion recognition [49,50]. An emotional state of the learner may suggest a modification in the presentation style and to be more interactive for effective tutoring. Use of emotion recognition is also getting prevalent in gaming where his emotional state governs a user’s interactions. Exergames (or gamercizing) frameworks [51,52], and other interactive and cloud-based gaming are becoming emotional-aware to provide a more immersive gaming experience [53,54]. The use of emotion recognition is also applicable in law for detecting and perceiving the intentions of the suspect [55] and in monitoring for risk prevention [56] and for smart health-care [57].

This work primarily explores the use of popular ML algorithms to recognize the intensity of emotion in combination with popular feature extraction algorithms. Following is a brief explanation of the major contributions of this comparative study:

- Three feature extraction algorithms, Gabor Features, Histogram of Oriented Gradients (HOG) and Local Binary Pattern (LBP), have been used and compared on five popular databases (B DFE, CK, JAFFE, and one of our own).
- Three popular ML algorithms, SVM, RF, and kNN were used for emotion intensity recognition.
- A comparative study and implementation of algorithms for measuring facial emotions and their intensities based on the different AUs (Action Units) are presented.
- The highest accuracy achieved for LBP combined with SVM shows that the intensity of emotions can be further used for real-world applications such as crime prediction systems and drowsy driver detection in vehicles to prevent fatalities [58].

The paper includes an in-depth literature review which discusses recent works in the area of facial emotion intensity recognition and is presented as Section 2. The current challenges and motivation behind this research are also discussed in Section 2. Section 3 gives a brief insight into the experiment along with the techniques that were used. We pay particular attention to the face detection techniques and emotion recognition algorithms in this section. The different correlations between Action Unit (AU) intensities and the description of the method for calculating intensities of the emotions based on facial expressions are described in Section 3.1. AUs can be understood as the specific parts (or units) of a face which come into action while a face depicts emotion. This section also discusses the gold-standard categorization of AUs, popularly used in emotion detection and recognition systems. The accuracy achieved from various experiments and the comparative study is presented in Section 4. Finally, the paper is concluded with a discussion on insights gained from the experiments and future work in Section 5.

## 2. Literature Review

Facial emotion recognition has been used for a variety of applications such as the identification of Autism and Schizophrenia, detection of a drowsy driver [59], identifying abnormalities in early stages of Alzheimer’s disease or schizophrenia, and for crime prediction systems. Before we discuss compare the prevalent works, it is important to understand the datasets that were used to train the recognition algorithms by popular works. Several databases exist that have been used, and are summarized in Table 1 which has been reproduced from [60]. It is also important to understand that several algorithms pose limitations. As an example, the Viola-Jones algorithm can be used along with Haar feature selection and AdaBoost training algorithm for remarkable detection of the eye region and nose bridge region with the limitation that it is only effective for the frontal images and can hardly cope with a 45${}^{\circ }$ face rotation [61].

Furthermore, we studied various works where emotion recognition was reported as an aggregate score of all emotions or expressions and summarized our findings in Table 2. We also studied works that attempt to measure the emotional intensity of AUs, and therefore, facial emotion. Here, we look at some of the works in a little more detail that reported accuracies for individual emotion detection. Jens et al. experimented to find the expression leakage in relation to the emotional intensity with a database created by capturing images of 21 participants [68]. The study was based on three emotions of happiness, sadness, and fear. The results of the study showed that there was facial expression leakage between the posed and genuine emotions. These *leaks* were measured by finding the intensity differences between the emotions. The participants were shown video footage and were told to enact genuine and posed emotions for the experiments. The study was limited to participants from an academic background with an average age of 19.4.

Hess et al. measured the intensity of emotional facial expression for the emotions of happiness, sadness, anger, and disgust, posed by two men and women [83]. The expressions depicted had six levels of emotional intensities. The accuracy achieved were 85.3% (anger), 79.3% (disgust), 97.9% (sadness), and 91.8% (happiness). The method included collecting and pre-processing the colored images into high-quality grayscale images. This study was conducted using a set of 5 pre-defined intensity of expressions of 20%, 40%, 60%, 80%, and 100%. The authors concluded the study by providing evidence that the perceived intensity of the underlying emotion varied linearly with the physical intensity of expressions.

Biele et al. performed an experiment where the intensity of happiness and anger were measured using static and dynamic images (animations) [23]. Anger was noted to be more intense than the extreme happy emotion. Also, the intensity results for dynamic images were higher than that of the static images. Gender differences also had an impact on the results, e.g., a difference in intensity was observed for anger in males and both emotions among females. They used the 2 (Subject Sex) · 2 (Actor Sex) · 2 (Emotion) · 2 (Stimuli Dynamics) ANOVA method for finding the intensity of emotions. A need for a new methodology for measuring the intensity of emotions to have a better insight into how men and women processed different emotional information was stressed upon in this work.

Another work experimented the perceived emotion by measuring the intensities of the emotions [84]. The basis of this analysis relied entirely on the fact that facial expressions conveyed rich information concerning the state of mind. Researchers have argued that neuromotor programs of facial expression patterns might be able to serve as the building blocks of emotion communication [84,85,86,87]. The interaction pattern was observed behaviorally for emotion intensity ratings, and neutrally for functional magnetic resonance imaging activation in the amygdala (a roughly almond-shaped mass of gray matter inside each cerebral hemisphere, involved with the experiencing of emotions), as well as fusiform and medial prefrontal cortices. However, this behavior was observed only for mild-intensity expressions.

Delannoy et al.’s experiments were based on the image-based approach for the three ranks of intensities, low–medium–high [88]. They performed one-against all SVMs for training classifiers. They used only 68 images from 10 subjects from the CK+ dataset and used ten-fold cross-validation. However, the drawback in their approach was that multi-class classifiers approach assume that the rankings of intensities were independent of each other. Hence the relation between labels was not well employed for performance enhancement. Furthermore, even with the classification of expressions in three intensity rankings, the AU must still be extracted from the image sequence as a feature representation.

Several other researchers performed experiments to conduct more accurate expression intensity estimation results [78,89,90,91]. The authors employed the training data with known intensity labels for these works. These intensity labels were categorized into two sections, discrete rank representations [88,89], and continuous value representations [92]. Once the label intensities were employed, the expression intensity degree was predicted and validated more accurately. The drawback of these approaches was that multiple images or an image sequence were required to estimate the intensity of emotion and. Also, sufficient images were required for estimation in a sequence-based approach.

Littlewort et al. performed their experiment by conducting estimation of the intensity rankings for facial expressions by using SVMs [93]. The authors used the distances to the SVM hyperplanes to estimate the rankings of emotion intensities. Chang et al. performed a manifold learning approach discrimination for recognizing facial expressions and used the distances of the manifold to determine the intensities of the emotions [94]. However, the distances to the classification boundaries in the feature space may not necessarily reflect how neutral/strong was an expression and hence, concluded inaccurate results.

Rudovic et al. proposed a method using intrinsic topology of multidimensional continuous facial affect data. An ordinal manifold first modeled the data. The topology was then used for the (H-CORF) Hidden Conditional Ordinal Random Field. Later, it was used for dynamic ordinal regression [95]. This was done to constrain H-CORF parameters to lie on the ordinal manifold. The resulting model attained simultaneous dynamic recognition and intensity estimation of facial expressions of multiple emotions. This method was used for both posed and spontaneous expressions. They also tested their model on databases such as BU-4DFE, CK, and CK+. All this research on the previously applied techniques and these facts provide the evidence that brings us to the conclusion that more research is required for the accurate detection of emotions with intensities.

### Current Challenges and Motivation

Even with a higher accuracy of emotional intensity measurements, practical real-time systems face a lot of problems. These problems are primarily related to time resolution, low-level emotion recognition (facial expressions captured with low peak frequencies), scarcity of the available databases for research-related intensity measurement, face and angle variation, illumination variations, and non-alignment of the faces. The research involving facial emotion recognition is divided into two categories: (i) image-based and (ii) video-based. Image-based recognition uses static frames for the recognition, while the video-based method includes dynamic frames for recognition. A lot of work is needed to address the problems mentioned above. Low-level emotion recognition is one such problem, since measuring the facial intensity of emotion is directly related to human–machine interaction (HMI), where robots can interact more naturally if they know the intensity of the emotion of the person they are communicating with. Security, surveillance, biometrics, and patient monitoring are a few other problems that have not been explored much. Majority of the work concentrates on the emotions depicted at the peak-level of the intensity, neglecting the emotions depicted at lower intensity levels as they are comparatively difficult to recognize. Measuring the low-level frame intensity is a challenge as the available databases lack the discriminating features.

Comprehensive standards were designed by Paul Ekman and Friesen to subdivide each emotion into several special AUs which is popularly known as the Facial Action Coding Units (FACS). These categorizations are considered to be a gold standard for emotion detection and recognition systems. An insight into the relevant literature in the field reveals that the examination of the accuracy of intensity predictions of 5 basic emotions from spontaneous/posed facial expressions has been highly ignored. Besides, the attention has been primarily paid to the examination of the factors that affect the perception of the category of emotion (the one that leads to expression analysis such as happiness/sadness/fear/anger), neglecting the intensity. Naturally, this does not mean that research on the accuracy of intensity prediction is less critical. With advances in technology, providing an input image and classifying it into one of the six emotions (5 basic, and one neutral) is proving to be insufficient. Hence, more attention needs to be given to the intensity of the five basic emotions to judge and understand human emotions for HMI, medical, and biometrical applications. Over the years, psychological research has shown that understanding the dynamics of the expressions is equally essential to understand human emotion. In other words, emotion dynamics is primarily related to emotion intensity variations in spatial and temporal domains. The future where robots successfully understand human emotions through intensity is far away since much work remains to be done. This underlying problem was the primary motivation to follow the AUs and their intensity to solve futuristic issues.

## 3. Methodology

**Figure 2:**
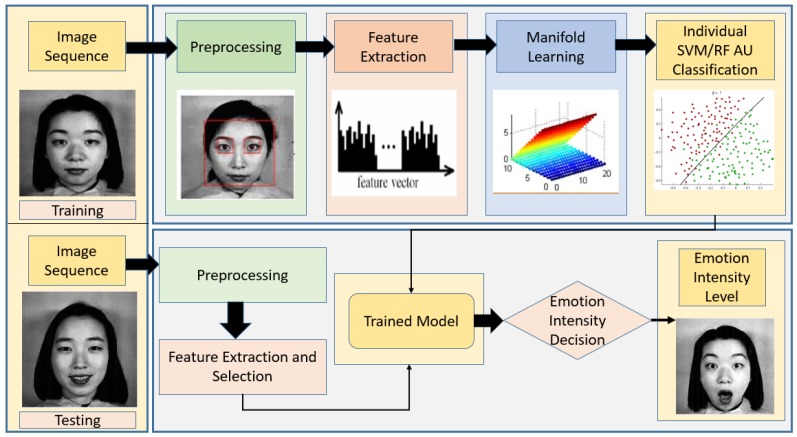
Generalized Architecture for Intensity of Emotion Recognition.

In this work, a comparison between the feature extraction techniques and the classification algorithms is presented to find the best combination that can be used for emotion intensity recognition. Figure 2 shows an overview of the experiment in the form of a generalized architecture, where training and testing layers are shown in detail. The first layer, called the training layer, has the following stages:

1. Image input and sequencing.
2. Pre-processing such as masking, scaling, converting into grayscale, and noise reduction.
3. Feature extraction algorithms such as LBP, Gabor, HOG are used, and a final feature vector is created using concatenation.
4. To remove the unwanted features, dimensionality reduction was used
5. Classification algorithms such as SVM, RF, and kNN were used to classify the AUs.

The second layer, testing layer, primarily has two stages. First, similar to the training stages, image sequencing, pre-processing, and feature extraction & selection was performed. Second, these features were passed through the trained model and finally an emotion intensity decision was made based on the AUs. Before we further discuss the intricate details of our comparative study, it is important to understand that this comparative study evaluates multiple techniques at both the intermediary step of feature extraction as well as the final step of classification.

HOG and LBP both create histograms to express features. HOG uses gradients to build spatial and orientation cells and assembles histograms of these gradients using overlapping spatial blocks while LBP considers a neighborhood block and computes and normalizes the histograms by converting the binary-threshold code to an integer. On the other hand, Gabor features are extracted using Gabor filters and use frequency patterns of regions of interest to extract features for segmentation and texture analysis. Gabor filter uses functions that relate filter size, oscillation frequency/phase, and orientation. Although technically Gabor filter is closest to the human visual perception system, LBP is known to be computationally simpler and work better in various illuminations. HOG, on the other hand, comes with the advantage of using different block sizes and number of histogram bins, unlike LBP.

The ML techniques we used in this work include kNN, SVM, and RF. While SVM is known to have a generalization ability by mapping inputs non-linearly to higher dimensional feature spaces through its capability of separating training data with a hyperplane. kNN, a type of instance-based learning, involves the neighbors deciding the class (among k classes) a specific data point belongs to. Closest neighbors are assigned using popular methods such as Euclidean or Hamming distance. RF is a collection of several decision trees which do not need linear features or even features that interact linearly. These three classification algorithms are known to perform well for high-dimensional spaces as well as a large number of training samples. Each of these algorithms works well under specific circumstances, kNN for noisy data, SVM for linearly inseparable data, and RF for categorical features. Due to these specific features, we chose to use these methods. All these techniques have been widely used in the literature, as discussed in Section 2. This section further discusses the essential details of the experiment performed.

### 3.1. AU Intensity Feature Extraction and Correlation Analysis

**Figure 3:**
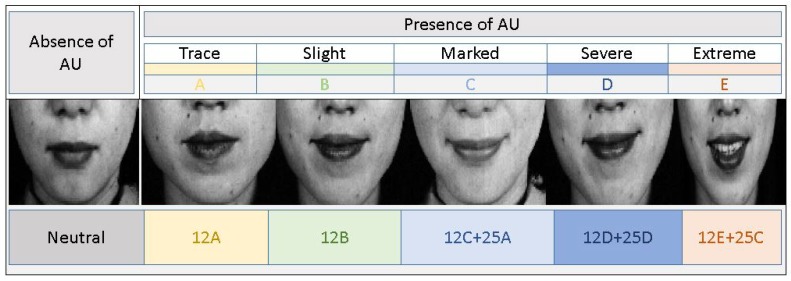
Relation between scale of evidence and intensities of facial action units.

This section consists of the description of the observed AU intensity feature extraction model, which consists of facial image registration and representation, dimensionality reduction, feature extractors, and classifications as shown in Figure 3. In this paper, we represent and capture the semantic AUs relations, as well as the correlation between the intensities of the AUs. This is done to measure the intensities of facial emotions more robustly.

**Figure 4:**
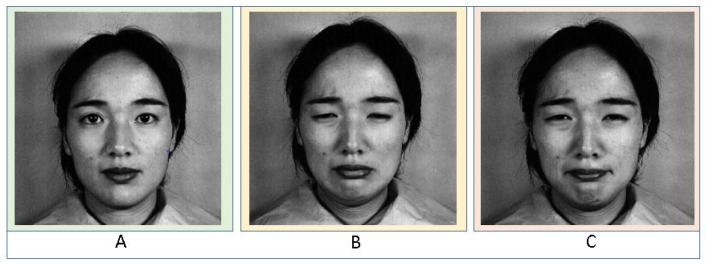
AU Combination: (**A**) AU12 occurs alone; (**B**) AU15 occurs alone; (**C**) AU12 and AU15 occur together—non-additive.

Due to the variety, the dynamics of facial actions, and the ambiguity, it is challenging to measure the intensities of AUs in a single frame. Mostly, databases are created with posed and spontaneous expressions, where it is a challenge to measure the intensity of spontaneous expressions as they occur more randomly. AUs significantly occur in combinations, where they are not always additive. This implies that the occurrence of an AU can be different than its original standalone type. A perfect example is shown in Figure 4, where AU12 occurs alone in Case A and the lip corner are pointing straight-slightly upwards. In Case B, AU15 lip corners appear a bit angled towards the ground, and in Case C, both co-occur, they are non-additive and hence, recognizing that emotion and intensity of the emotion become more difficult.

**Figure 5:**
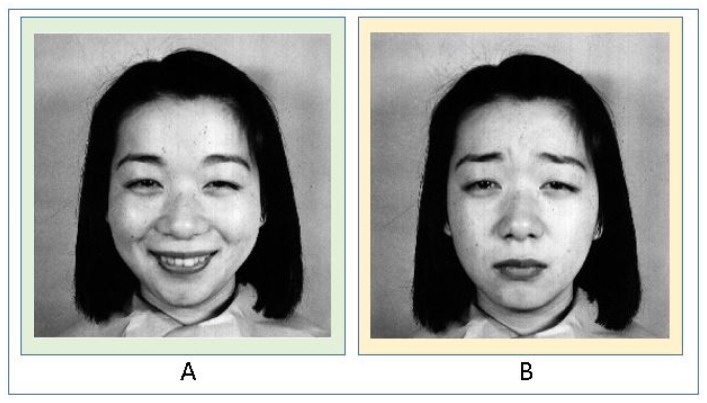
AU Combination: (**Case A**) AU6 + AU12 + AU25, (**Case B**) AU4 + AU15 + AU17.

FACS manual gives an insight into the inherent relationship between the AUs that can produce the required information for measuring and analyzing the emotional intensity. The manual mentions that the inherent relationships can be subdivided into two classes—the class of mutual exclusions and the class of co-occurrences. Here, the class of co-occurrences is a class of groups of AUs which generally and most frequently appear together to give meaning to the depicted facial emotions. For example, AU6 + AU12 + AU25 suggest “happy” while AU4 + AU15 + AU17 depict “sad”. In the case of mutually exclusive AUs, the FACS manual provides alternative rules. Mutually exclusive AUs rarely occur together in spontaneous emotions in day-to-day life. FACS mentions that it is difficult to demonstrate two AUs such as AU25 (lips apart) and AU24 (pressed lips) together at all. This suggests that the mutually exclusive cases are very much possible, but with very low probability. There are still few limitations to the co-occurrence class in terms of intensity levels of emotion AUs. For example, when AU6 (raised cheeks) occur with AU12 (lip corner puller) as shown in Figure 5, both the AUs present a high/low-intensity level of one another.

#### 3.1.1. Face Registration and Representation

This step aligns the data of a similar kind, such as input facial images plus the referenced facial images. Landmark points were used to mark to represent the important location of facial components such as the eyes, nose, and lips. To obtain the landmark points, an averaging solution was used, and this averaging was done over the entire training data set. The images are finally masked for the extraction of important facial regions and re-sized to 128 × 108 pixels. After this step, three well-known algorithms such as Histogram of Oriented Gradient, Gabor Features, and LBP were used for feature extraction for the reason that they are highly capable of representing the appearance-based information accurately.

#### 3.1.2. Feature Extraction through Gabor Features

Gabor features are comparative to the human visual system because of their frequency and orientation representations. A 2D Gabor feature, in the spatial domain, is a Gaussian kernel function which is modulated by a sinusoidal plane wave. These filters can be generated by one major wavelet by rotation and filter. These are the best among the other existing relevant image features such as the edge orientation histograms, box filters. In our experimentations, we extracted magnitudes on 96 × 96 images sizes using the directions of eight wavelets and scales of nine so that the Gabor wavelengths vary from the range of 2 to 32 pixels in half octave intervals. Although the resulting feature vector has 9 × 8 × 96 × 96 = 663,552 components, not all of them are useful. In fact, in our experiment, a very small number of informative components are selected. To perform Gabor analysis, first the eye centers are located, and then the images are aligned accordingly. This alignment is done by performing transformation, rotation, and scaling. This is a typical procedure for 2D images for registration. Normalization is done using manual determination of landmarks. This is done to preclude any misalignment effects from the registration schemes.

#### 3.1.3. Local Binary Pattern Method

The LBP method is based on a texture descriptor that is useful in extracting features from any textured image. We used an LBP for extracting facial features that are used for estimating the intensity of the emotion depicted in the image. The LBP is non-overlapping and uniform when applied to an image. Initially, a user specified number of uniform blocks are used to segment the image. For each patch on the image, the LBP matches the center pixel to its surrounding neighboring pixel to generate an *LBP* value. Equations (1) and (2) mentioned below are used for computation of *LBP*, where *N* represents the adjacent pixels, *k* is the neighboring size, and C is the center pixel. For this research, we have considered the value of *k* = 8.

(1) $$LBP\left(N,C\right)=\sum_{k=0}^{7}P\left(N_{k}-C\right)2^{k}$$

(2) $$\left[y\right]=\left\{\begin{array}{cc} 1 & \mathrm{for}\;y\geq 0\\ 0 & \mathrm{for}\;y<0 \end{array}\right.$$

The function *LBP* (*N*, *C*) (from Equation (1)) uses the P (Nk-C) as seen in Equation (2), generates a 1 or a 0 depending on the difference between the center pixel and the neighbor. Figure 4 shows an example of a neighboring pixel with their intensity values. Later the differences are calculated considering the center pixel. Equation (1) is used for the transition from difference matrix to Bit String Matrix (is a sequence of 0’s and 1’s). The most important step in LBP is that the starting position must be arbitrarily chosen for calculation. This is done by unwrapping the bit string and decoding it.


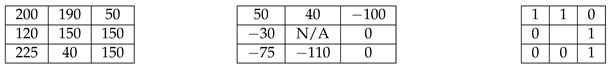


The number of bit string pattern within a patch is counted to create a feature vector that is used in a distance measure. For an 8-bit string, there are a total of 256 possible bit strings. Furthermore, for simplification of the process, the string is either considered to be uniform or non-uniform. A string is considered to be uniform when its bits, parsed in a circular sequential manner, has a shift of values two or fewer times. Similarly, a string is non-uniform when its bits have changed more than two times. e.g., consider the string 00011110. Here only two shifts occur. One between the third and fourth position and one between the seventh and eighth position. Out of the total 256 patterns, only 58 are uniform. For every patch of an image, a histogram is created which is composed of 59 bins. All the 58 uniform patterns are assigned to those 58 bins in the histogram, where each bin stores the frequencies of the patterns. The one bin which is left (59th bin) keeps an account of all the non-uniform patterns found in the patch. Furthermore, all the histogram vectors from patches are concatenated to represent a histogram representing the features extracted by the LBP.

#### 3.1.4. Histogram of Oriented Gradient Features

This method was initially used in the human detection area, further used as object detectors and finally, they are now used for analyzing and representing the facial emotions. The descriptor HOG can quickly and efficiently describe the local shape and appearance of objects by counting the occurrences of gradient orientations in a localized portion of the images. In this study, the images are divided into small cells, and for every single cell the histogram of the gradient is calculated. This is done to represent the spatial information of the face image. For every image, in our study, 48 cells are constructed out of every image by building a cell with 18 × 16 pixels. A horizontal gradient filter [−1 0 1] was applied with 59 orientation bins in the study. Final step done was the concatenation of all the HOG representations of each cell to form a HOG feature vector (size of 2832 (48 × 59)).

#### 3.1.5. Dimensionality Reduction

High-dimensional features of an image make the analysis of the samples more complicated in the real-world applications where ML and pattern recognition algorithms are used. When extracting and selecting features, several features extracted are redundant and should be removed. e.g., in ML, univariate feature selection is made to avoid the use of redundant features for training. Literature review above has shown that facial expression and intensity of those expressions are embedded along a low dimensional manifold in a high-dimensional space. In our study, we have implemented nonlinear techniques for preserving the local information which is further useful in the classification of the intensity of facial emotions and their representation. Manifold learning is a technique which presumes that the sample data points are collected from a low dimensional manifold and embedded into a high-dimensional space. Quantitatively, Consider, a set of points (6), find a set of points (7), such that $y_{i}$ represents $x_{i}$ efficiently.

(3) $$x_{1}\ldots x_{n}\in R^{D}$$

(4) $$y_{1}\ldots y_{n}\in R^{D}\left(d\backslash D\right)$$

The Laplacian eigenmap algorithm was used in our study to reduce the dimensionality of the data. Furthermore, the high-dimensional data was mapped to a 29-dimensional space. The basics of the algorithm are to map the closest points of the high-dimensional space into the close points of the low dimensional space. For problem-solving, the generalized eigenvector problem is applied. Further to describe the embedded d-dimensional Euclidean space the first d eigen vectors in correspondence to the first d eigen values are used. Spectral regression algorithm was used to find a projection function which can map the high-dimensional data, in our study the HOG, Gabor features, and LBPs into low dimensional space.

#### 3.1.6. Classification

AU intensity classification was performed using SVM, RF, and kNN classifiers after reducing the feature vectors. SVMs analyze the data and are used for pattern recognition. They construct a hyperplane or a set of hyperplanes which are further used for regression and classification problems. Discriminative hyper planes are found by the SVM classifiers including the highest margin for dividing the data that belongs to the different classes. However, several kernel types can affect the efficiency of the SVM classifiers. In our study, we used the C=6 AU intensity levels of each of the following AUs. The strategy considered is the one-against-one strategy where C(C−1)/2 binary discriminant functions are defined, one for each possible pair of classes. The Gaussian RBF kernel was used. In our study, each AU and each frame were considered individually since it is an appearance-based approach. The results are also largely affected by the face region alignment.

### 3.2. Databases Considered

Training of ML algorithms depend on the type and size of database used. A low number of images in a database that is being used for training, can cause under-fitting. To counter the issue, we need a large database for training the available ML algorithms. The estimations and results are remarkably affected by the use of larger databases over the smaller ones; hence, for more accurate results a database is required in abundance. Emotion classification and their intensity estimations require a vast and varied dataset for validation and testing. The images used for intensity estimation and recognition of emotion are spontaneous and posed.

- Posed Datasets: Popular for capturing extreme emotions. The disadvantage is the artificial human behavior.
- Spontaneous Datasets: Natural human behavior. However, it is extremely time-consuming for capturing the right emotion.

Hence, a close relationship exists between the models used for the intensity of facial emotions and the databases used. Mainly five databases CK [62,63], JAFFE [64], B-DFE and an in-house dataset of 200 images (20 images of each of the basic emotions considered) taken in an uncontrolled environment through a web camera. Here is a brief description of each database:

1. JAFFE: The database contains 213 images of 7 facial expressions (6 basic facial expressions + 1 neutral) posed by 10 Japanese female models. Each image has been rated on 6 emotion adjectives by 60 Japanese subjects. (Posed – no AUs present)
2. DISFA: In this database the images were acquired using PtGrey stereo imaging system at high resolution (1024 × 768). The intensity of AU’s (0–5 scale) for all video frames were manually scored by two human FACS experts. The database also includes 66 facial landmark points of each image in the database. (Spontaneous—AUs present (AU1, AU2, AU4, AU5, AU6, AU9, AU12, AU, 15, AU17, AU20, AU25, AU26))
3. CK: Subjects in the released portion of the Cohn-Kanade AU-Coded Facial Expression Database are 100 university students. They ranged in age from 18 to 30 years. Sixty-five percent were female, 15 percent were African-American, and three percent were Asian or Latino. Subjects were instructed by an experimenter to perform a series of 23 facial displays that included single AUs and combinations of AUs. (Posed: AUs present (AU1, AU2, AU4, AU5, AU27))
4. BU-3DFE: Includes 100 subjects with 2500 facial expression models. The BU-3DFE database is available to the research community (e.g., areas of interest come from as diverse as affective computing, computer vision, human computer interaction, security, biomedicine, law-enforcement, and psychology). The database contains 100 subjects (56% female, 44% male), ranging age from 18 years to 70 years old, with a variety of ethnic/racial ancestries, including White, Black, East Asian, Middle-east Asian, Indian, and Hispanic Latino. (Posed: AUs present (AU1, AU2, AU4, AU5, AU6, AU9, AU12, AU, 15, AU17, AU20, AU25, AU26))
5. Our own: Dataset consists of 200 images (20 images of each of the 5 basic emotions considered) taken in an uncontrolled environment through a web camera. The reason for collecting the dataset in an uncontrolled environment was that the intensity of facial emotions is to be measured in an everyday uncontrolled environment for practical real-time applications. The dataset consists of probe and gallery images which are taken over a month. There are in total 200 probe images and 100 gallery images from the same subjects of different emotions which are used for validation of the algorithm purposes. The images are taken in different angles and lighting conditions to see the effect of these factors on our proposed model. (No AUs present)

It should be noted that the AUs labeled by each database were different. Hence, comparison between posed and spontaneous images was not possible for all databases. The only comparison would be between DISF and B-DFE; however, B-DFE is a 3D image database and features extracted from those images unlike DISF, which had 2D images. Hence, a comparison between those two would not be a fair comparison. The feature vector of a 2D image was in the form [width, height, 3] and the feature vector in the 3D image dataset was of the form [width, height, depth, 3], i.e., both feature representations would be different and hence, not comparable. A possible way was to perform feature extraction by passing 3D volumes through a pre-trained 3D network/algorithm, or to perform 2D feature extraction on each slice of the volume and then combine the features for each slice, using PCA to reduce the dimensionality. However, this would impact the accuracy. Therefore, such a comparison was not presented.

## 4. Results

### 4.1. Recognition and Reliability Measures

Recognition rate and the Intra-Class Correlation (ICC) values were exploited to evaluate the proposed automated AU intensity measurement in our study. The statistical index, i.e., the ICC has a range from 0 to 1.

(5) $$\;ICC=\frac{BMS-EMS}{BMS+(k-1)\times EMS}$$

(6) $$\;EMS=\frac{ESS}{\left(k-1)\times (n-1\right)}$$

(7) $$\;BMS=\frac{BSS}{n-1}$$

It is also the measure of conformity for our data set since it has multiple targets. This study is basically where n participants are being judged by k number of judges. In our study, we assume *n* = 6 and *k* = 2. The purpose of using ICC is because it is preferred over the Pearson correlation between measurement and judges. The ICC shows the proportion of total variance between the targets. Here, BMS = Between target Mean Squares, EMS = Residual Mean Squares which are defined by the ANOVA (Analysis of Variance).

### 4.2. Result Analysis Based on Intensity of Emotions

The previous section discusses the feature extraction techniques we implemented for measuring AU intensities in facial emotions. The three techniques implemented include the LBP, Histogram of Oriented Gradient Features, and Gabor Features. These are followed by classification techniques such as Support Vector Machine, Random Forest and Nearest Neighbor Classifiers. Given all the image observations, we implement the network for measuring the intensity of emotion recognition for each AU. The results are presented in Table 3.

As shown in Table 3, the best results were achieved with the *LBP* with the nearest neighbor classifier while using all three features. This is because it models static relationships between the AU intensities. All the values in the table are percentage accuracy in detection. AUs which were not present for certain cases, have been indicated as NA (Not Applicable) while zeros indicate that the AU was present, but accuracy was 0 indicating that it was not recognized at all.

For observations in images, which are not very accurate, improvements are seen in features of Gabor wavelets using the random forest classifiers. Table 4 shows the performance of individual features when combined with popular ML algorithms. Besides, a correlation analysis for the AUs was done, for which we have listed a correlation matrix especially for the action units 1 and 2 in the Table 5. This matrix is a relation between the AU1 and AU2. The intensity dependency between both the AUs is proportional to each other. High of AU1 results in a high probability of AU2 and vice-versa. When AU2 is at level 0, AU1 probability is 0.982 and when the intensity at AU2 is level “3” AU1 probability at level “3” is 0.88. By calculating such AU dependency relationships between the action units, the ICC and the accuracy for various algorithms improved. Although not shown in the table, but we would like to mention that the accuracy increased from 68.32% to 71.95% for Random Forest when the AU dependency relationship was used while extracting HOG features. Similarly, for Gabor features, the accuracy increased from 79.11% to 82.13% for when the nearest neighbor algorithm was used. Since the AU intensity inference phase and the feature extraction phase are independent of each other, higher accuracy is achieved. Table 4 also shows that LBP performs best when used with SVM.

We also performed a comparison of our work with a few other works. As shown in Table 6, we noted that a few recent works [60,81] had used similar features (HOG and Gabor in both; LBP in one) and the DISFA database. Therefore, to have a fair comparison, we applied our approach. It is clear from Table 6 that the proposed method performs far better than other works while using the same feature selection methods and database. Table 7 presents the characteristics of the comparative state-of-the-art methods listing the databases, and feature extraction & ML methods used in each work. It should be noted that one of the primary differences between our work and other recent works is the use of a greater number of databases for training the ML algorithm while using similar feature extraction algorithm. Although the average accuracy and ICC percentages improved for all feature extraction methods, it is noteworthy that the highest improvement was seen with LBP when the SVM model was trained using 5 databases, as seen in Table 6.

**Figure 6:**
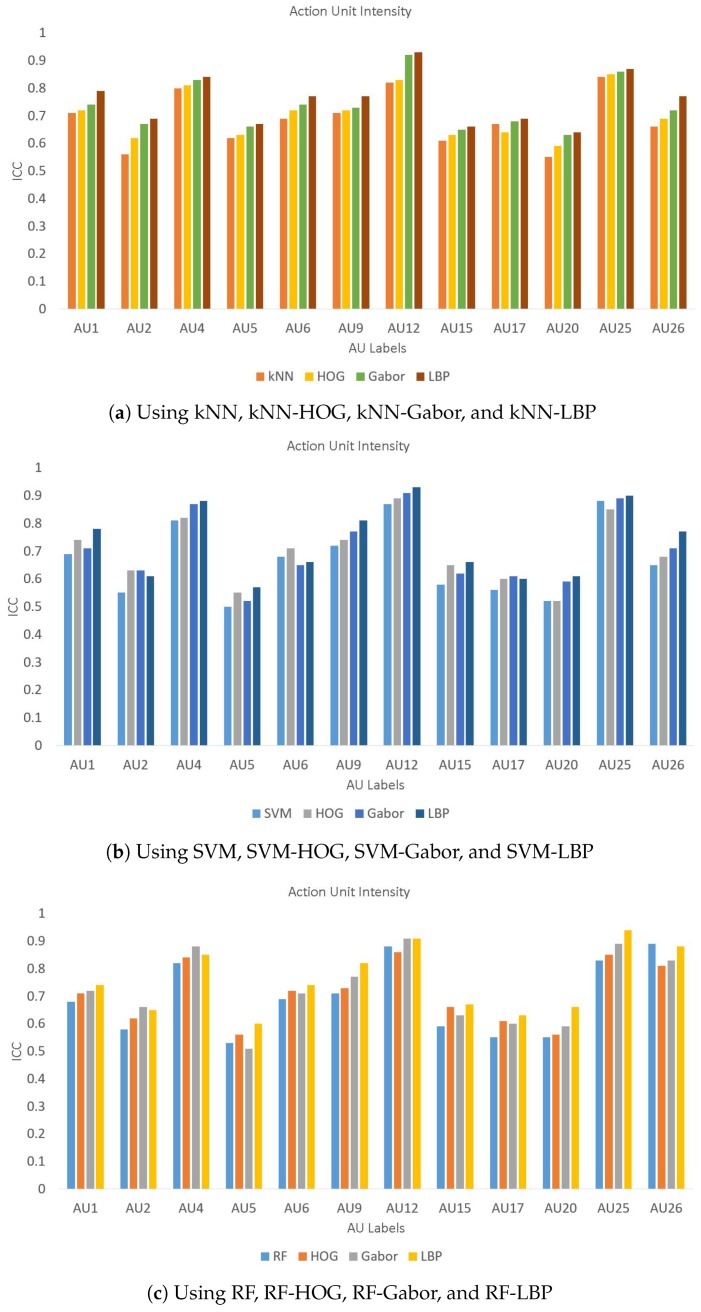
Comparison of AU intensity Labels on Database.

**Figure 7:**
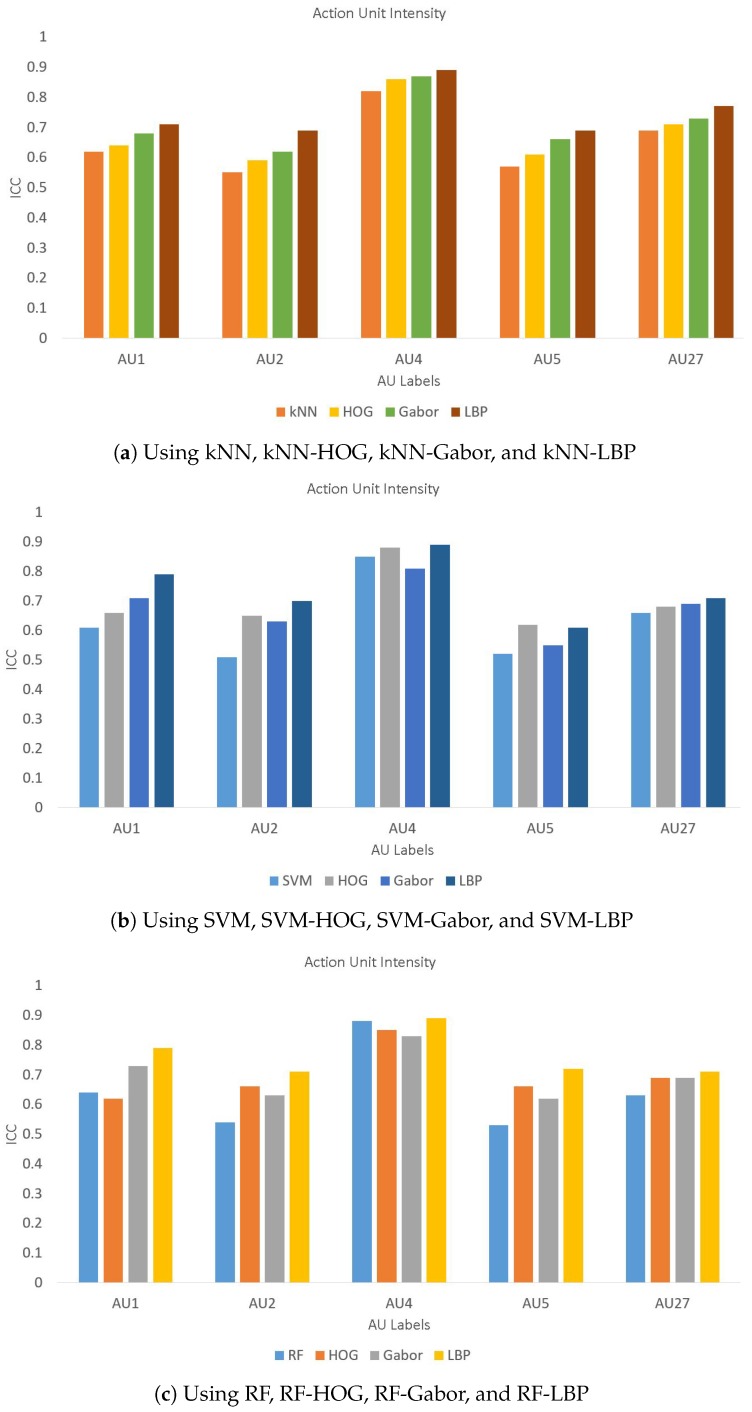
Comparison of AU intensity Labels on Database.

**Figure 8:**
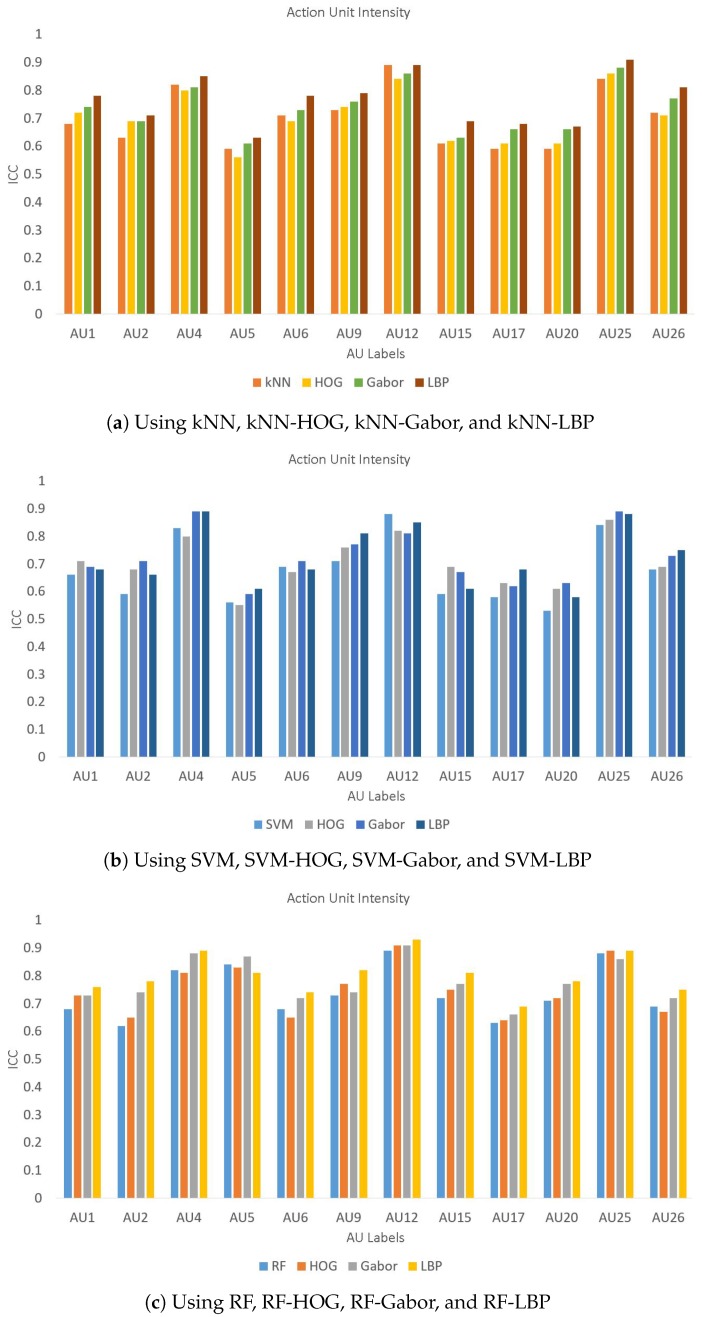
Comparison of AU intensity Labels on Database.

Furthermore, we also evaluated the performance of the proposed method for a few other databases including JAFFE, CK, B-DFE, and our dataset of 200 images. These results are presented in Table 8 and clearly shows that LBP performs better for the first three databases while the performance is quite close to the best performance of Gabor for the CK and databases. A few of these results are also presented using bar charts for a better visual comparison in Figure 6, Figure 7 and Figure 8 for databases, respectively. It is evident from these figures that LBP gives better results for almost all AUs and gives the best results when combined with SVM. Therefore, we conclude that the LBP feature extraction method, when used with SVM, works best for facial emotion intensity recognition.

In conclusion, it is evident from Table 3, LBP-kNN detects almost all AUs with high accuracy (>94%) while other techniques show this level of accuracy only for few AUs. Therefore, we LBP-SVM will recognize almost all emotions at all intensity levels better than the other studied techniques. It should also be noted that the average accuracy of detection of intensity of emotion decreases for all emotions with an increase in intensity; however, LBP+SVM still performs better than Gabor-SVM and HOG-SVM on average.

Nevertheless, in real-world applications, accuracy is also dependent on various other factors such as the image quality, environment it was captured in (controlled, uncontrolled), angle of the face, age of the person (fine lines on the face can make huge difference in accuracies), and lighting conditions. Also, accuracy differs from men to women, since women tend to express emotions more vividly than men. All these factors will come into consideration for emotion intensity as well as emotion detection for real-world face recognition systems and might significantly affect the performance of any technique.

## 5. Conclusions and Future Work

AUs are popularly used for measuring the facial emotion intensities from facial expressions. Use of an adequate amount of data is required for training and testing classifiers for its best performance in terms of accuracy. Majority of the databases consist of posed facial expressions or the emotion labels. Hence our research was focused on using the publicly available databases which have AUs that are annotated on a 6-point intensity scale. The experimentation was performed for both spontaneous and posed facial emotion intensity recognition, where we conclude that AU intensity is not always reliable and accurate in the case of spontaneous facial expressions. This happens due to the ambiguity and dynamic nature of the facial emotions when spontaneous expressions are taken into consideration. For measuring the intensity of emotions, it is not only required to improve the accuracy of feature extraction algorithms but also exploiting the facial actions. It is these spatiotemporal facial action interactions with synchronized and coherent actions that provide a full facial display. In our work, we presented a probabilistic model to calculate the ICC values and accuracies among the dynamic and semantic AU intensity levels. Also, AU intensity recognition is accomplished by integrating the images systematically with the proposed model. The accuracies for various algorithms (LBP, HOG, and Gabor) indicate that LBP achieves the highest accuracy in most cases. As a future work, in neural networks several hidden layers could be added to specifically handle each challenge in the spontaneous intensity of emotion recognition such as the head tilt and angle. 

## Figures and Tables

**Table 1:** Popular databases for measuring intensity of emotions (Reproduced from [60]). (Type: P: Only Posed, S: Only Spontaneous, SP: Spontaneous and Posed).

Database	Database Description	Emotion Description Intensity	Type
Cohn-Kanade (CK) [62]	100 multi-ethnic subjects, 69% female, 31% male (age: 18–50) with frontal and $30^{\circ }$ view	23 series of facial display, available, AU-coded face database (single and combination)	P
Extended CK (CK+) [63]	123 multi-ethnic subjects, extension of CK	66/123 subjects considered, spontaneous smiles, onset to peak coded	SP
JAFFE [64]	Japanese Female Facial Expression, Grayscale images with 10 subjects	6 basic emotions + neutral, 2–4 samples per expression, available	P
Bosphorus	105 subject, 44 female, 61 male	2D/3D AU-coded, pose, and illumination variations, available	P
BU-3DFE [65]	Binghamton University 3D Facial Expression, multi-ethnic, 56 female and 44 male (age: 18–70), 2500 samples	4 intensity levels, 6 basic emotions + neutral, available	P
RU-FACS	Rochester/UCSD Facial Action Coding System, 100 subjects	AU-FACS coded, private database with 33 AUs, unavailable	SP
NVIE	Natural Visible and Infrared (IR) facial Expression, Visible and IR imaging, 215 students (age: 17–31)	temporal analysis for face data, basic 6 expressions, available	SP
MMI	25 multi-ethnic subjects, 12 female, 13 male (age: 20–32)	Onset, offset, apex temporal analysis, single + combined AUs, available	SP
DISFA [60,66,67]	Denver Intensity of Spontaneous Facial Actions, 12 female, 15 male, 130,000 video frames	Intensity of 12 AUs coded, available	S
Belfast Induced Emotion	Three set of tasks, lab-based emotion induction tasks	Intensity plus emotion, trace style rating of intensity and valence	S
PAINFUL DATA	UNBC-McMaster Shoulder Pain Expression Archive Database, 200 video sequences, 66 female, 63 male	Pain related AUs coded, available	S

**Table 2:** Comparative study of accuracy achieved for intensity of emotions.

Work	Technique	Database	Accuracy	Drawbacks
[69]	Iterated Closest Point (ICP), PCA	355 images	92.00%	Low accuracy *
[70]	Geometric-based approach, Haar feature selection technique (HFST)	FRGC v2	97.00%	Unsuitable for real-time applications
[71]	K-means clustering with back-propagation	CK	98%	Unsuitable for real-time applications
[72]	AAM, Lucas-Kanade, BPNN	BU-3DFE	83.80%	Low accuracy
[73]	Feature Distribution Entropy, Euclidean distances between 83 3D facial feature points, Adaboost	BU-3DFE	95.1%	Unsuitable for real-time applications
[74]	Distance vectors, neural network	BU-3DFE	91.30%	Low accuracy
[75]	Principal Component Analysis	FRGC v1	95%	—
[42]	Stochastic Neighbor Embedding, Gabor wavelet, SVM	JAFFE	58.70%	Low accuracy, small database
[76]	Local Binary Patterns (LBP)	10 Volunteers	89.60%	Low accuracy, small database
[77]	Gradient-based ternary texture patterns, SVM	CK	97.10%	—
[78]	Gaussian curvature, Gabor wavelets, shape index, SVM	Bosphorus, (2902 images)	63.10%	Only 25 AUs from still images considered
[79]	Facial data generator, Hercules engine	Webcam data	89.60%	Low accuracy, slow
[80]	Spectral regression, SVM	18 min-video	92%	Limited data
[81]	Dynamic Bayesian Network (DBN), Adaboost (ADB)	DISFA	72.77% (DBN), 69.31% (ADB)	Low accuracy, AU extraction and intensity inference phases independent
[82]	Conditional random Field Model	DISFA, FERA2015	70% (DISFA), 50% (FERA)	Limited data, low accuracy

* Low accuracy indicates it is low for real-world applications.

**Table 3:** AU Intensity Measurement Results—HOG, Gabor and LBP (Acc: Accuracy Percentage).

	**HOG**													
**#**		**AU1**	**AU2**	**AU4**	**AU5**	**AU6**	**AU9**	**AU12**	**AU15**	**AU17**	**AU20**	**AU25**	**AU26**	**AVG**
1	ICC	0.71	0.68	0.8	0.55	0.67	0.76	0.82	0.69	0.63	0.61	0.86	0.69	0.70
2	Acc	87.22	71.49	89.48	92.76	89.01	94.18	91.89	77.1	97.15	79.3	96.14	97.74	**88.62**
3	L = 0	88.13	89.67	90.17	87.28	88.97	78.56	93.47	95.17	88.14	95.58	89.66	84.36	89.09
4	L = 1	87.31	77.41	79.36	74.15	68.36	69.48	88.54	87.79	84.58	74.69	78.34	77.14	78.92
5	L = 2	84.26	59.68	36.74	68.45	77.96	85.14	39.62	47.27	53.99	77.4	69.35	65.72	63.79
6	L = 3	77.74	66.03	82.23	91.18	66.89	93.78	86.79	73.8	84.57	78.45	79.88	84.33	80.47
7	L = 4	55.63	59.68	69.74	66.13	64.58	78.69	67.48	61.34	61.79	79.77	78.49	77.41	68.39
8	L = 5	33.19	18.97	92.36	49.89	0	54.36	0	NA	16.89	NA	68.36	69.44	40.34
	**Gabor**													
**#**		**AU1**	**AU2**	**AU4**	**AU5**	**AU6**	**AU9**	**AU12**	**AU15**	**AU17**	**AU20**	**AU25**	**AU26**	**AVG**
1	ICC	0.82	0.87	0.89	0.59	0.81	0.87	0.8	0.79	0.76	0.69	0.96	0.87	0.81
2	Acc	88.9	91.74	83.69	96.27	88.74	93.18	96.78	98.36	71.49	68.98	85.47	82.89	**87.20**
3	L = 0	99.1	97.86	97.84	95.36	99.67	97.36	99.87	96.87	98.48	98.36	99.55	99.68	98.33
4	L = 1	68.15	26.68	84.15	79.36	88.36	84.16	91.89	77.98	89.74	85.69	87.48	89.79	79.45
5	L = 2	77.89	74.59	89.61	84.67	80.57	84.36	86.74	75.06	82.71	70.11	78.31	68.03	79.38
6	L = 3	79.36	61.92	56.98	65.69	66.26	69.93	55.53	76.25	73.81	82.11	89.02	61.93	69.89
7	L = 4	64.69	55.81	53.67	59.57	61.26	60.72	48.69	0	70.14	85.97	68.14	60.25	57.40
8	L = 5	33.69	64.56	59.57	55.79	0	15.79	0	NA	0	NA	97.82	93.67	42.08
	**LBP**													
**#**		**AU1**	**AU2**	**AU4**	**AU5**	**AU6**	**AU9**	**AU12**	**AU15**	**AU17**	**AU20**	**AU25**	**AU26**	**AVG**
1	ICC	0.89	0.88	0.74	0.85	0.83	0.84	0.77	0.78	0.91	0.74	0.82	0.87	0.82
2	Acc	97.81	98.17	94.09	96.07	96.87	96.74	98.99	98.065	98.77	95.67	95.89	98.88	**97.16**
3	L = 0	85.69	81.02	68.19	79.6	98.24	92.16	77.4	91.8	90.56	96.3	77.49	80.45	84.90
4	L = 1	53.64	27.73	44.69	60.13	42.94	33.02	59.23	67.14	66.98	78.09	74.08	76.31	57.00
5	L = 2	68.42	61.08	78.61	98.07	58.41	60.79	82.66	87.9	61.27	71.08	63.78	55.09	70.59
6	L = 3	59.67	68.07	82.34	57.09	63.87	90.27	83.44	67.07	56.65	77.4	59.77	61.79	68.95
7	L = 4	47.83	80.17	86.49	97.11	84.03	66.08	72.11	77.6	95.17	96.79	93.37	88.27	82.08
8	L = 5	90.81	49.67	56.47	33.96	0	84.17	55.37	NA	0	NA	87.28	66.98	52.47

**Table 4:** Summary of Accuracies for various Feature Types.

#	Feature Type	kNN	RF	SVM
1.	HOG Feature	68.64	71.95	88.62
2.	Gabor Wavelets	82.13	85.6	87.20
3.	LBP	92.11	96.33	97.16

**Table 5:** Correlation Matrix for Action Units.

#	AU Intensity	AU1 = 0	AU1 = 1	AU1 = 2	AU1 = 3
1.	AU2 = 0	0.982	0.0097	0.0063	0.0079
2.	AU2 = 1	0.60	0.322	0.078	0.051
3.	AU2 = 2	0.324	0.290	0.313	0.131
4.	AU2 = 3	0.124	0.0837	0.158	0.88

**Table 6:** Comparison with state-of-the-art methods.

#	Works	HOG		Gabor		LBP	
		Acc	ICC	Acc	ICC	Acc	ICC
1.	[60]	79.14%	0.70	85.71%	0.77	81.54%	0.69
2.	[81]	81.08%	0.7016	86.60%	0.7834	—	—
3.	Our Work	88.62%	0.70	87.20%	0.81	**97.16%**	0.82

**Table 7:** Characteristics of the state-of-the-art comparative methods.

#	Works	Databases for Training	Feature Extraction Techniques	Machine Learning Techniques
1.	[60]	DISFA	HOG, Gabor, LBP	SVM
2.	[81]	DISFA	HOG, Gabor	DBN
3.	Our Work	B-DFE, JAFFE, CK & In-house	HOG, Gabor, LBP	SVM, kNN, RF

**Table 8:** Performance of proposed method for different Databases.

#	Databases	HOG	Gabor	LBP
1.	200 images	96.17%	97.08%	99.11%
2.	JAFFE	97.23%	98.07%	99.10%
3.	CK	98.69%	98.71%	98.14%
4.	B-DFE	97.31%	98.64%	98.02%
